# Supplementary material for: Exact projected entangled pair ground states with topological Euler invariant
Source: Nat Commun. 2025 Jan 2;16:284. doi: 10.1038/s41467-024-55484-4 (PMC11695689; doi:10.1038/s41467-024-55484-4)
Supplement: Supplementary file 1 — Supplementary Information [file 41467_2024_55484_MOESM1_ESM.pdf]

Supplementary Information for "Exact projected entangled pair ground states with topological Euler invariant"

Thorsten B. Wahl,[1,*] Wojciech J. Jankowski,[1] Adrien Bouhon,[1,2] Gaurav Chaudhary,[1] and Robert-Jan Slager[1,†]

[1] *TCM Group, Cavendish Laboratory, Department of Physics, J J Thomson Avenue, Cambridge CB3 0HE, United Kingdom*

[2] *Nordita, Stockholm University and KTH Royal Institute of Technology, Hannes Alfvéns väg 12, SE-106 91 Stockholm, Sweden*

(Dated: December 9, 2024)

In this Supplementary Information, we provide details on the quantum geometric properties of our non-interacting PEPS and connect the quantum Fischer information with the quantum Cramer-Rao bound for ideal Euler bands.

Supplementary Note 1: Quantum geometry in the free fermion limit

In the following, we highlight the ideal quantum geometrical properties due to the flatness of the bottom bands. The flatness is crucial, as it allows for Hamiltonians which are sums of local projectors and therefore have (macroscopically degenerate) ground states at energy $E = 0$. The quantum metric [1–3],

$$g^{\chi}_{ij} = \text{Tr}_{\text{occ}}[(\partial_{k_i}\widehat{P})(\partial_{k_j}\widehat{P})], \tag{1}$$

is defined as a trace over momentum-space projectors $\widehat{P} = \sum_{n=1,2} |u_n(\mathbf{k})\rangle \langle u_n(\mathbf{k})|$ of occupied Bloch states $|u_n(\mathbf{k})\rangle$, with the momentum components $k_i, k_j = k_1, k_2$. The flat Euler bands saturate the quantum-geometric bounds, due to the non-zero Euler invariant χ [2, 4], between the quantum volume elements ($\sqrt{\det\, \mathbf{g}^{\chi}}$) and the Euler curvature ($\sqrt{\det\, \mathbf{g}^{\chi}} = |\text{Eu}|$) across the entire momentum space. Upon integrating, we thus retrieve a quantum volume which is a multiple of 2π,

$$\text{Vol}\; \mathbf{g}^{\chi} \equiv \oint \sqrt{\det\, \mathbf{g}^{\chi}}\; \text{d}k_1 \wedge \text{d}k_2 = 2\pi|\chi|, \tag{2}$$

showcasing the ideal non-Abelian quantum geometry [2, 4]. Upon introducing interactions, the many-body quantum metric in the space of twisted boundary conditions can reflect the topological nature of many-body Euler ground states as we also demonstrate in the non-interacting limit, see below. Using central relations of quantum metrology [5–7], the ideal condition physically manifests itself through the non-triviality of the quantum Fischer information (QFI). That is, we retrieve a metrological quantum Cramer-Rao (QCR) bound [5] on the realizable model measurements, see the next Section, which could be directly executed in quantum simulators or synthetic three-level systems [8].

We now concretize these points more formally. Consistently with the Plücker formalism for multi-band quantum geometry introduced in Ref. [2], we can define the Fubini-Study metric ($\text{d}s^2 = 1 - |\langle u_1(\mathbf{k}) \wedge \ldots \wedge u_n(\mathbf{k}) | u_1(\mathbf{k}+\text{d}\mathbf{k}) \wedge \ldots \wedge u_n(\mathbf{k}+\text{d}\mathbf{k})\rangle|^2$) in the set of occupied Bloch bands $\{|u_n(\mathbf{k})\rangle\}$ [1],

$$\text{d}s^2 = g^{\chi}_{ij}(\mathbf{k})\text{d}k_i\text{d}k_j \tag{3}$$

where g^{χ}_{ij} is the quantum metric in the Euler flat bands, and the Einstein summation convention was assumed. With both of the Euler bands $n = 1, 2$ occupied ('occ'), we can correspondingly write the metric as

$$g^{\chi}_{ij}(\mathbf{k}) = \sum_{n}^{\text{occ}} \frac{1}{2}\Big[\langle\partial_{k_i}u_n(\mathbf{k})|\,\widehat{Q}\,|\partial_{k_j}u_n(\mathbf{k})\rangle + \text{c.c.}\Big] = \langle\partial_{k_i}u_1(\mathbf{k})|u_3(\mathbf{k})\rangle\langle u_3(\mathbf{k})|\partial_{k_j}u_1(\mathbf{k})\rangle + \langle\partial_{k_i}u_2(\mathbf{k})|u_3(\mathbf{k})\rangle\langle u_3(\mathbf{k})|\partial_{k_j}u_2(\mathbf{k})\rangle\,, \tag{4}$$

where $\widehat{Q} = \sum_m^{\text{unocc}} |u_m(\mathbf{k})\rangle\langle u_m(\mathbf{k})| = |u_3(\mathbf{k})\rangle\langle u_3(\mathbf{k})|$ is the projector onto unoccupied ('unocc') band(s); here, $m = 3$. In the second equality, we used the fact that the eigenvectors representing the Euler bands are chosen real, as here, the Bloch Hamiltonian $H(\mathbf{k}) = \sum_{i=1}^{3} E_i(\mathbf{k})\,|u_i(\mathbf{k})\rangle\langle u_i(\mathbf{k})|$ is a real symmetric matrix. The quantum metric is manifestly real and symmetric, by definition Supplementary Equation (4).

Alternatively, we can rewrite the metric in terms of the projector onto the unoccupied band as,

$$\begin{aligned} g^{\chi}_{ij} = \text{Tr}_{\text{occ}}[(\partial_{k_i}\widehat{Q})(\partial_{k_j}\widehat{Q})] &= \text{Tr}_{\text{occ}}(|\partial_{k_i}u_3(\mathbf{k})\rangle\langle\partial_{k_j}u_3(\mathbf{k})| + |u_3(\mathbf{k})\rangle\langle u_3(\mathbf{k})|\partial_{k_i}u_3(\mathbf{k})\rangle\langle\partial_{k_j}u_3(\mathbf{k})| \\ &\quad + |u_3(\mathbf{k})\rangle\langle\partial_{k_i}u_3(\mathbf{k})|u_3(\mathbf{k})\rangle\langle\partial_{k_j}u_3(\mathbf{k})| + |u_3(\mathbf{k})\rangle\langle\partial_{k_i}u_3(\mathbf{k})|\partial_{k_j}u_3(\mathbf{k})\rangle\langle u_3(\mathbf{k})|) \\ &= \langle\partial_{k_i}u_3(\mathbf{k})|\partial_{k_j}u_3(\mathbf{k})\rangle - \langle\partial_{k_i}u_3(\mathbf{k})|u_3(\mathbf{k})\rangle\langle u_{3,\mathbf{k}}|\partial_{k_j}u_{3,\mathbf{k}}\rangle = \langle\partial_{k_i}u_3(\mathbf{k})|\partial_{k_j}u_3(\mathbf{k})\rangle \end{aligned} \tag{5}$$

where the last equality follows from the reality condition. We now us the fact that the third Bloch band defines a normalized vector field: $\hat{\mathbf{n}}(\mathbf{k}) \widehat{=} |u_3(\mathbf{k})\rangle$, as follows from the spectral decomposition of the Hamiltonian.

In terms of the momentum-space vector $\hat{\mathbf{n}}$, the quantum metric in a three-band Euler Hamiltonian reads [9]

$$g^{\chi}_{ij} = (\partial_{k_i} \hat{\mathbf{n}}) \cdot (\partial_{k_j} \hat{\mathbf{n}}), \tag{6}$$

which obtains an inequality [4],

$$\sqrt{\det \, \mathbf{g}^{\chi}} \geqslant |\mathrm{Eu}|. \tag{7}$$

Additionally, from inequality between arithmetic and geometric means, we directly obtain,

$$\mathrm{Tr} \, \mathbf{g}^{\chi} \equiv g^{\chi}_{11} + g^{\chi}_{22} \geqslant 2\sqrt{g^{\chi}_{11} g^{\chi}_{22}} \geqslant 2\sqrt{g^{\chi}_{11} g^{\chi}_{22} - (g^{\chi}_{12})^2} \equiv 2\sqrt{\det \, \mathbf{g}^{\chi}} \geqslant 2|\mathrm{Eu}|, \tag{8}$$

where we used the symmetry of the (real) metric tensor $g^{\chi}_{12} = g^{\chi}_{21}$.

In the considered model, the metric elements read:

$$g^{\chi}_{11} = \frac{8 - 3\cos k_1 - 3\cos(k_1 + k_2) - \cos(k_1 - k_2) - \cos(k_1 + 2k_2)}{8(3 + \cos k_1 + \cos k_2 + \cos(k_1 + k_2))^2}, \tag{9}$$

$$g^{\chi}_{22} = \frac{8 - 3\cos k_2 - 3\cos(k_1 + k_2) - \cos(k_1 - k_2) - \cos(2k_1 + k_2)}{8(3 + \cos k_1 + \cos k_2 + \cos(k_1 + k_2))^2}, \tag{10}$$

$$g^{\chi}_{12} = g^{\chi}_{21} = \frac{2 - 2\cos k_1 \cos k_2 + \sin k_1 \sin k_2}{4(3 + \cos k_1 + \cos k_2 + \cos(k_1 + k_2))^2}, \tag{11}$$

which directly obtains the quantum volume [4],

$$\sqrt{\det \, \mathbf{g}^{\chi}(\mathbf{k})} = \frac{-3 + \cos k_1 + \cos k_2 + \cos(k_1 + k_2)}{4\sqrt{2}(3 + \cos k_1 + \cos k_2 + \cos(k_1 + k_2))^{3/2}}, \tag{12}$$

as well as

$$\mathrm{Tr} \, \mathbf{g}^{\chi}(\mathbf{k}) = \frac{16 - 3\cos k_1 - 3\cos k_2 - 6\cos(k_1 + k_2) - 2\cos(k_1 - k_2) - \cos(2k_1 + k_2) - \cos(k_1 + 2k_2)}{8(3 + \cos k_1 + \cos k_2 + \cos(k_1 + k_2))^2}. \tag{13}$$

On the contrary, the Euler curvature in the model is given by the following expression

$$\mathrm{Eu}(\mathbf{k}) = \frac{-3 + \cos k_1 + \cos k_2 + \cos(k_1 + k_2)}{4\sqrt{2}(3 + \cos k_1 + \cos k_2 + \cos(k_1 + k_2))^{3/2}}. \tag{14}$$

We note that, analytically, an inequality $\mathrm{Tr} \, \mathbf{g}^{\chi}(\mathbf{k}) \geqslant 2|\mathrm{Eu}(\mathbf{k})|$ holds, on substituting the individual quantum metric matrix elements to the bound between the determinant and trace. The equality of the determinant (quantum volume) and the Euler curvature follows trivially by inspection, as the analytical expressions for both quantities are identical across the entire momentum space.

Moreover, beyond the single-particle context, we can consider many-body quantum metric $g_{ij}(\theta)$ defined in terms of the twist angles $\theta = (\theta_1, \theta_2)$ and the twisted boundary conditions [10],

$$\psi(\{x_i + L_1\}, \{y_i\}) \equiv \langle \{x_i + L_1\}, \{y_i\} | \psi \rangle = e^{i\theta_1} \psi(\{x_i\}, \{y_i\}), \tag{15}$$

$$\psi(\{x_i\}, \{y_i + L_2\}) \equiv \langle \{x_i\}, \{y_i + L_2\} | \psi \rangle = e^{i\theta_2} \psi(\{x_i\}, \{y_i\}), \tag{16}$$

where $i = 1, 2, \ldots, N_{\mathrm{tot}}$ are particle labels, x_i and y_i the sets of coordinates of fermions i, and L_1, L_2 denote the cell lengthscales, on which the twisted periodic boundary conditions were imposed on the many-body state.

In terms of the twist angles, the many-body quantum metric reads

$$g_{ij}(\theta) = \mathfrak{Re} \, \langle \partial_{\theta_i} \psi(\theta) | \, (1 - \hat{P}_{\theta}) \, | \partial_{\theta_j} \psi(\theta) \rangle \,, \tag{17}$$

with the projector onto the many-body ground state $\widehat{P}_\theta = |\psi(\theta)\rangle \langle\psi(\theta)|$. At the zero twist angle $\theta = (0,0) \equiv \mathbf{0}$, in the free-fermion limit, we retrieve a many-body bound,

$$g_{ij}(\mathbf{0}) = \frac{1}{L_1 L_2} \sum_{\mathbf{k}} \text{Tr } \mathbf{g}^{\chi}(\mathbf{k}) \geqslant \frac{4\pi A}{L_1 L_2} |\chi|, \tag{18}$$

where A is the area of the unit cell of the system. However, we note that unlike in the case of the determinant bound providing an ideal condition on Euler bands, this many-body bound does not saturate in the considered models, as it reduces to the trace bound in the free particle limit. In other words, here, the strong inequalities rather than equalities hold within the proposed models.

Supplementary Note 2: Quantum Fischer information and quantum Cramer-Rao bound of the ideal Euler bands

We further comment on the structures present in the models and their relation to quantum Fisher information (QFI) and the quantum Cramer-Rao (QCR) bound [5, 6]. Namely, we derive a non-Abelian QCR bound, which is induced by Euler topology and the Euler bands satisfying an ideal condition. The QCR bounds are of central relevance for quantum metrology [6].

To define the QFI in the context of this work, we consider a two-parameter family of single-particle states $|\psi_n(\mathbf{k})\rangle$, parametrized by $\mathbf{k} \equiv (k_1, k_2) \in T^2$, where T^2 denotes a two-torus. Consistently with the models introduced in the main text, at every point $\mathbf{k}$ of the parameter space, we consider a three-state system. We take a spectral decomposition of the density matrix of the single-particle states at given $\mathbf{k}$-point, $\rho \equiv \sum_n \lambda_n |\psi_n(\mathbf{k})\rangle \langle\psi_n(\mathbf{k})|$. The QFI matrix for single-particle operators $\widehat{r}_1$, $\widehat{r}_2$ (conjugate to k_1, k_2), then reads [6],

$$F_{ij}[\rho] \equiv \sum_{m,n:\lambda_m+\lambda_n \geqslant 0} 2\frac{(\lambda_m - \lambda_n)^2}{\lambda_m + \lambda_n} \langle\psi_m(\mathbf{k})| \widehat{r}_i |\psi_n(\mathbf{k})\rangle \langle\psi_n(\mathbf{k})| \widehat{r}_j |\psi_m(\mathbf{k})\rangle \,. \tag{19}$$

In translationally symmetric contexts, if the parameters $\mathbf{k}$ were to be identified with momenta, then $\widehat{r}_1$, $\widehat{r}_2$ represent position operator components defined along the lattice vectors. We moreover recognize: $\widehat{r}_i \sim i\partial_{k_i}$, i.e. $-i\partial_{k_i}\rho = [\rho, \widehat{r}_i]$, and hence, $-i\langle\psi_m(\mathbf{k})| \partial_{k_i}\rho |\psi_n(\mathbf{k})\rangle = \langle\psi_m(\mathbf{k})| [\rho, \widehat{r}_i] |\psi_n(\mathbf{k})\rangle = (\lambda_m - \lambda_n)\langle\psi_m(\mathbf{k})| \widehat{r}_i |\psi_n(\mathbf{k})\rangle$. Therefore, for pure states, where in the context of this work we can consider single particle in the third band within the corresponding three-state problem, i.e. $\rho = |\psi_3(\mathbf{k})\rangle \langle\psi_3(\mathbf{k})| = |u_3(\mathbf{k})\rangle \langle u_3(\mathbf{k})|$; the QFI matrix reduces to the quantum metric (see the main text),

$$F_{ij}[\rho] = 4g^{\chi}_{ij}(\mathbf{k}). \tag{20}$$

The QCR bound [5, 6] for the two-parameter measurements can be captured by the covariance matrix Σ with [6, 8],

$$\Sigma(\widehat{\mathbf{k}}) \geqslant \frac{1}{M} F^{-1}[\rho], \tag{21}$$

where M is the number of the repetitions of measurements [6–8]. The covariance matrix for an unbiased estimator $\widehat{\mathbf{k}}$ for the two-parameter family $\mathbf{k} = (k_1, k_2)$ under a set of positive operator-valued measurements (POVM), Π_p, such that $\sum_p^{N_p} \Pi_p = 1$, $\Pi_p \Pi_{p'} = \Pi_p \delta_{pp'}$, with $N_p \geqslant 3$; is defined as [6, 7],

$$\Sigma_{ij}(\widehat{\mathbf{k}}) = \langle \delta k_i \delta k_j \rangle \equiv \sum_p k_i k_j \text{Tr}[\rho \Pi_p] - k_i k_j, \tag{22}$$

where $\langle \ldots \rangle \equiv \text{Tr}[\rho(\ldots)]$. For ideal bands, as in the introduced model, the Euler curvature determines the quantum-metrological bound at every point of the parameter space as,

$$\sqrt{\det\, \Sigma(\widehat{\mathbf{k}})} \geqslant \frac{1}{M\sqrt{\det\, \mathbf{g}^{\chi}(\mathbf{k})}} = \frac{1}{M|\text{Eu}(\mathbf{k})|}, \tag{23}$$

where the first inequality follows from the derivation of Ref. [7], and the second equality is realized in the models introduced in our work.

Beyond the demonstrated quantum-metrological manifestations, the realized ideal condition for the Euler bands opens avenues for exotic fractionalization of the excitations in the topological bands, and offers a platform for exploring further deeper connections to the many-body quantum metric under twisted boundary conditions.

---

* tw344@cam.ac.uk
† rjs269@cam.ac.uk

[1] J. Provost and G. Vallee, Commun. Math. Phys. **76**, 289 (1980).
[2] A. Bouhon, A. Timmel, and R.-J. Slager, Quantum geometry beyond projective single bands (2023).
[3] P. Törmä, Phys. Rev. Lett. **131**, 240001 (2023).
[4] S. Kwon and B.-J. Yang, Phys. Rev. B **109**, L161111 (2024).
[5] S. L. Braunstein and C. M. Caves, Phys. Rev. Lett. **72**, 3439 (1994).
[6] J. Liu, H. Yuan, X.-M. Lu, and X. Wang, J. Phys. A: Math. Theor. **53**, 023001 (2019).
[7] B. Mera, A. Zhang, and N. Goldman, SciPost Phys. **12**, 018 (2022).
[8] M. Yu, X. Li, Y. Chu, B. Mera, F. N. Ünal, P. Yang, Y. Liu, N. Goldman, and J. Cai, Nat. Sci. Rev. , nwae065 (2024).
[9] W. J. Jankowski, A. S. Morris, A. Bouhon, F. N. Ünal, and R.-J. Slager, arXiv:2311.07545 (2023).
[10] Q. Niu, D. J. Thouless, and Y.-S. Wu, Phys. Rev. B **31**, 3372 (1985).
